# Emission of harmful gases from animal production in Poland

**DOI:** 10.1007/s10661-021-09118-7

**Published:** 2021-05-17

**Authors:** Kamila Mazur, Kamil Roman, Witold Jan Wardal, Kinga Borek, Jan Barwicki, Marek Kierończyk

**Affiliations:** 1 Institute of Technology and Life Sciences, Branch in Warsaw, Warsaw, Poland; 2 Institute of Wood Sciences and Furniture, Warsaw University of Life Sciences, Warsaw, Poland

**Keywords:** Emissions, Ammonia, Natural ventilation, Air exchange

## Abstract

The aim of the study was to present the scale of greenhouse gas emissions from animal production, and to provide test results from different housing systems. In three free stall buildings, two with slurry in deep channels and one with cattle in cubicles staying on shallow litter concentration of ammonia and carbon dioxide were measured in summer season by using dedicated equipment from Industrial Scientific Research. Air exchange was calculated on the base of balance carbon dioxide method. This method was used in order to estimate the air flow rate. Concentrations of ammonia and CO_2_ were measured as the base for air exchange and ammonia emission rates. Ammonia emissions were product of ammonia concentration and air exchange rate. Temperature and relative humidity were measured to establish microclimate conditions in buildings tested to show the overall microclimatic situation in buildings. Differences between ammonia emission rates were observed in both housing systems. The highest ammonia emission rate was equal to 2.75 g·h^−1^·LU^−1^ in well-ventilated cattle barn with the largest herd size.

## Introduction

Milk and meat production are finally balanced with an environmental and animal welfare conditions to minimize negative influence for the environment. Major amount of nitrogen are leaching from livestock production to the environment.

According to the inventories, agriculture is a significant source of greenhouse gases (GHG) (Roman et al., 2019). In 2015, the EU agricultural sector emitted 3751 kt of ammonia and was responsible for 94% of total ammonia emissions (Crippa et al., 2018; EUROSTAT, 2020). Poland is one of the most important contributors to nitrogen atmospheric emissions in the Baltic Sea Region (EUROSTAT, 2020).

Moreover, significant amounts of harmful ammonia gas are derived from livestock production. Cattle are responsible for 70% of total greenhouse gas emissions (Philippe & Nicks, 2015).

There is a lack of data about ammonia emissions from cattle barns from central Europe. Ammonia emissions differ depending on climate zone, housing system, manure management (Baldini et al., 2016), type of feed (Bougouin et al., 2016) and animal breed.

Air temperature in the barn is the most important factor affecting ammonia emissions (Sanchis et al., 2019). Literature analysis shows that authors from across Europe describe the problem of harmful gases in connection with animal production. We have some works from Poland Herbut and Angrecka (2014) and Pietrzak (2006)—and abroad—Demmers et al. (1998), Dore et al. (2004), Jungbluth et al. (2001), Mohn et al. (2018) and Poteko et al. (2019).

Tied-up cattle barns were under observation using measurements of ventilation rate and concentration of harmful ammonia gas (Karłowski et al., 2008). The measurements were carried out of ammonia emissions from manure plate by using micrometeorological passive dosimetry method (Ferm et al., 2005; Marcinkowski, 2010).

There were prepared by Russian scientists’ table of harmful gas emissions, including methane and forms of nitrogen from different cattle housing systems in intensive production in cold climate (Gridnev et al., 2014) (Table 1).

**Table 1:** Results of model analysis of typical dairy farms with feed production on the farm

Housing system	Tied-up	Tied-up (2nd type)	Free stall	Free stall (2nd type)
Basic herd size cattle	100	100	200	200
	142	142	284	284
Milk yield, l	5000	5000	6000	6000
CH_4_ losses (eq.CO_2_), t	344.5	347.8	834.4	819.9
Emission CH_4_∙kg∙cow∙year^−1^	4.05	4.16	4.65	4.84
N_2_O losses (eq. CO_2_), t	663.0	645.3	1500.7	1329.0

Source: own elaboration based on Gridnev et al. (2014)

Ammonia emissions from systems with natural ventilation depend heavily on the efficiency of the ventilation system; the more effective it is, the greater the probability of higher emissions. Bougouin described negative impact of milk production on NH_3_ emission that milk yield had on NH_3_ emissions (Bougouin et al., 2016).

Demmers indicates that the CO_2_ balance method demands not only the presence of animals inside the building but also detailed knowledge about CO_2_ quantities. According to this information, carbon oxide could be a better tracer gas because of its features: its density is almost the same as the air and it can be measured by continuously working data analyzer, and is inertive enough and has low background concentration.

Table 2 shows the amounts of chosen GHG emissions according to Krawczyk and Walczak (2010). There were balance chambers used with steady thermal-humidity conditions and a steady air exchange rate. In this work, ammonia emissions tested from cattle barns with slurry and with solid manure in shallow boxes were presented.

**Table 2:** Gaseous emissions from housing systems of technological groups (kg year^−1^∙LU^−1^)

	_Housing system_					
	Littered straw	Littered sawdust	Deep litter straw	Deep litter sawdust	Without litter	Slotted
Dairy cows						
Water vapor	3456.4	3562.1	3732.6	3862.8	3956.4	x
Carbon dioxide	2664.8	2545.3	2989.4	2844.1	2764.8	x
CH_4_	108.4	112.91	123.53	126.32	119.2	x
N_2_O	0.032	0.045	0.062	0.073	0.416	x
Heifers						
Water vapor	3110.4	3456.1	3567.1	3595.9	3645.2	3723.7
Carbon dioxide	1944.6	1823.8	2078.3	1924.5	1998.2	2129.7
CH_4_	56.3	57.4	79.32	84.27	66.73	67.58
N_2_O	0.01	0.016	0.019	0.021	0.022	0.024
Calves						
Water vapor	x	x	1941.43	2059.2	X	2178.4
Carbon dioxide	x	x	1108.23	1046.3	x	987.8
CH_4_	x	x	21.2	24.47	x	19.6
N_2_O	x	x	0.002	0.004	x	0.006

Source: own elaboration based on Krawczyk and Walczak (2010)

## Methods

Determination of emissions from buildings with natural ventilation demands measurements of gas concentrations and air exchange rates. Also, CFD methods are available for ammonia emission modelling, but they still need to develop (Bjerg et al., 2013a, b; Yi Q et al., 2019a, b). In this study, levels of ammonia and carbon dioxide concentrations were tested both inside and outside the 3 boxed livestock buildings: one with shallow litter and two with slurry in deep channels.

Both gas concentration and air exchange rate should be measured, especially for naturally ventilated livestock buildings as determination of it is problematic. In such cases, tracer gas methods are used (as a type of balance method). Nosek et al. (2020) confirmed that tracer gas method is very useful for ventilation rate estimation.

For example, some researchers used CO_2_, SF6 or cryptone 85 as tracer gases (Müller et al. 2007; Kiwan et al., 2012).

Edouard et al. (2016) used tracer gas method as well as moisture balance method. Indicators of CO_2_ emissions by livestock animals and water vapor are not constant and depend on the animals, age and diet. In our study, the CO_2_ balance method was used.

The methods in our research consisted of the following stages:

1. Measurements of ammonia concentrations in few points inside cattle barns (S) by using gas concentration meters, made by company Industrial Scientific Co.
2. Estimation of air exchange rate (*V*) using validated method of carbon dioxide balance. For metabolic emission of carbon dioxide by one LU, average values were used W_CO2_ = 220 g·h^−1^·LU^−1^ according to the Institute of Zootechnics in Cracow.
3. Calculation of ammonia emission (*E*).

Ammonia emission (*E*) was equal product of air exchange rate (*V*) and ammonia concentration (*S*):

1 $$E=V\cdot S$$

where:

*E*—ammonia emission from building [g·h^−1^·LU^−1^],

*V*—air exchange rate in building [m^3^·h^−1^·LU^−1^],

*S*—average ammonia concentration from measurement points, reduced by the concentration of this gas in the air flowing into the cattle barn [ppm, converted into g m^−3^].

The ventilation rate was calculated using the carbon dioxide balance method from the equation:

2 $$V=\frac{{\mathrm{WCO}}_{2}}{{C}_{\mathrm{inside}}-{C}_{\mathrm{outside}}}\left[{\mathrm{m}}^{3}\cdot {\mathrm{h}}^{-1}\right]$$

where:

*V*—air exchange rate in building [m^3^·h^−1^·LU^−1^],

W_CO2_—metabolic emission of carbon dioxide by one LU [g·h^−1^·LU^−1^],

*C*_inside_—average CO_2_ concentration inside cattle barn—average from measurement points measured in particular time [ppm, converted into g·m^−3^],

*C*_outside_—average CO_2_ concentration in air inflowing into the building [ppm, converted into g m^−3^].

Finally, ammonia emission was equal:

3 $$E=\frac{{\mathrm{WCO}}_{2}}{{C}_{\mathrm{inside}}-{C}_{\mathrm{outside}}}\cdot S$$

where:

*E*—ammonia emission from building [g·h^−1^·LU^−1^]; other marks supra.

Additionally, temperature and relative humidity were measured using thermo-hygrometers.

The following measurement equipment was used:

- 4 multi-gas monitors for CO_2_ and NH_3_ concentrations. They were mobile, with own memories, type MX6, American producer Industrial Scientific,
- 4 thermo-hygrometers LB-710 (TH-5, TH-6, TH-7, TH-8), connected with concentrator LB-731 for data collecting.

## Results

A short characteristic of herd like herd size and system of removing manure is shown in Table 3. The annual milk yield was at the range from 7000 to 9500 l in the extra class for cows Holstein–Friesian breed. In two boxed cattle barns with slatted floors, the slurry was collected in deep manure channels and pumped out from them. Additionally, robotic manure scrapers were regularly removing the slurry from slatted floors making them more clear. In all buildings, natural light was from the windows in the walls and from roof ridge gap. Table 4 presents the statistical values of ventilation rates (air exchange rates) and estimated diurnal average ammonia emissions from cattle barns tested during the summer period (June–July). Temperature and air relative humidity were measured separately.

**Table 3:** General characteristics of tested objects

No	LU	Housing type	Ventilation system	Unitary cubage	Average milk yield of herd; litres∙cow^−1^ year^−1^	Manure removing system
3	50	Free stall boxed, shallow litter (straw), solid floor	Gravitational ventilation, air inflow through wall openings; outflow through roof ridge gap	107.8	7000	2 kg of straw per 1 LU, littered daily; hydraulic manure scrapers, twice a day
2	140	Free stall, boxed, without litter	Gravitational ventilation, air inflow through wall openings—mobile curtains; outflow through roof ridge gap	70.64	8500	Slurry in deep channels; robotic manure scraper 5 times per day
3	83	Free stall, boxed, without litter, slatted floor	Gravitational ventilation, air inflow through wall openings; outflow through roof ridge gap	74.00	9500	Robotic manure scraper 3 times per day

Source: own study

**Table 4:** Gaseous emissions from housing systems of objects tested (kg·year^−1^·LU^−1^)

No. of cowshed	Statistical value	Temperature inside	Relative humidity outside	Relative humidity inside	Air exchange rate (V)	NH_3_	NH_3_ emission*	CO_2_
		[°C]	[%]	[%]	[m^3^·h^−1^·LU^−1^]	[ppm]	g·h^−1^·LU^−1^]	[ppm]
1	Mean	23.92	73.86	73.3	262.2/203.2*	5.22/11.31*	1.73/2.64*	792/1132*
	Min	19.53	38.64	52.12	160/84.9	1/2*	0.2/0.27*	300/500*
	Max	27.3	96.7	87.4	3653.5/1826.7*	17/18*	3.6/37.03*	500/1733*
2	Mean	23.69	48.14	58.31	401.76	11.97	2.75	845.5
	Min	18.59	18.4	21.28	170.4	1	0.78	450
	Max	30.61	67.65	77.34	3784.3	23	3.73	1380
3	Mean	17.72	59.25	69.32	399.65	6.16	1.47	665
	Min	11.94	38.15	56.54	167.59	1	0.59	300
	Max	21.85	95.13	90.21	3687.1	19	6.95	1500
Recommendation (Collective work 2005)		Optimal 8–16	-	Optimal 70 max. 80	450	Max. 20		Max. 3000

^*^Day/night, source: own study

The obtained results of harmful gas emission which is ammonia depend on the effectiveness of the ventilation.

The highest level of ammonia emission was observed from cattle barn with deep slurry channels and with the highest ventilation rate which amounted 2.75 (g·h^−1^·LU^−1^). In contrary, the lowest emission 1.47 (g·h^−1^·LU^−1^) was observed in a cattle barn with the lowest cubage.

**Fig. 1:**
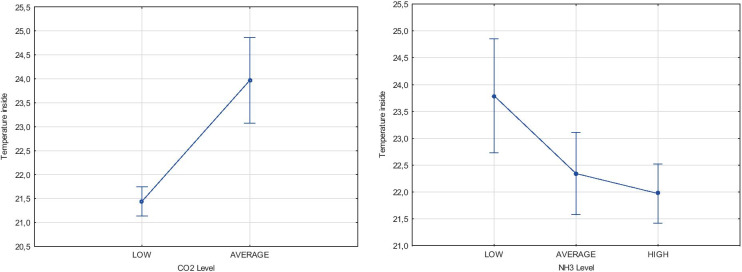
The results of temperature comparison concerning CO_2_ and NH_3_ levels. Source: own study

According to the above-presented table, the values of NH_3_ and CO_2_ emission levels were estimated. The established high, average and low levels of gas emissions were created, as multiple values of 7 ppm for NH_3_ and 1000 ppm for CO_2_. Created levels were dependent on the recommended limits of NH_3_ that equal 20 ppm and CO_2_ equal 3000 ppm. Using the estimated levels, correlation of environmental parameters in reference to the gas emissions from cattle houses was conducted. The ANOVA method was chosen as a tool for statistical analysis. During the statistical analysis, the temperature inside, relative humidity outside and humidity inside were correlated to the gas emissions. The results of temperature compared with the CO_2_ and NH_3_ levels were presented in Fig. 1.

**Fig. 2:**
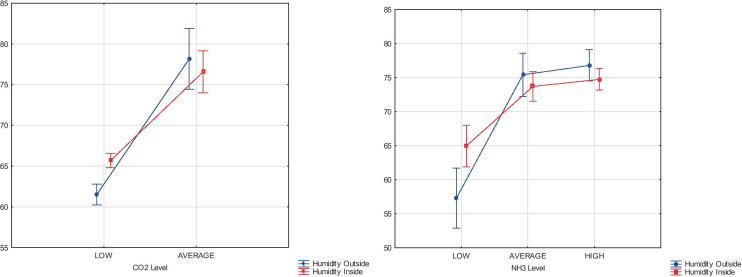
The results of relative humidity outside and relative humidity inside comparison with the CO_2_ and NH_3_ levels. Source: own study

During the statistical analysis, the expected marginal mean of temperature influence to CO_2_ and NH_3_ emission density was specified. In the case of temperature impact on the CO_2_ emission level, the significance value (*p*) was below than critical level of 0.05 (5%), and the statistical empirical value *F*(1, 846) = 27.494. The statistical analysis of the temperature influencing the NH_3_ emission level delivers that the significance level (*p*) was 0.01184 and the statistical empirical value *F*(2, 846) = 4.4595. The case of temperature impact on the CO_2_ emission level, inversely than the NH_3_ emission level, delivers the correlation. The obtained results were the basis for the Duncan tests that determine the temperature values to homogeneous groups. The analysis showed that each of the tested temperatures is in a different homogeneous group, which makes significant differences in the temperature impact on the level of CO_2_ emissions. The mean temperature for the low level of the CO_2_ emission was 21.5 °C, for the average level was close to the 23.2 °C, but high emission was not known. The correlation of relative humidity outside and relative humidity inside with the NH_3_ emissions from cattle houses was conducted. The estimated recommended levels of NH_3_ emissions were also used. The results of relative humidity outside and humidity inside correlation with the CO_2_ and NH_3_ levels were presented in Fig. 2.

It was statistically confirmed that the relative humidity outside and relative humidity inside had an influence on the CO_2_ and NH_3_ emission levels in both cases. Similarly, in both statistical analyses, the significance value (*p*) was below a critical level, which means that the correlation exists. The empirical value of statistics *F*(2, 845) during the relative humidity outside and relative humidity inside comparison with the CO_2_ was equal to 34.726, and the Wilks Lambs = 0.92405. In the case where the relative humidity outside and relative humidity inside were correlated with NH_3_, the empirical value of statistics *F*(4, 1690) = 17.507 and the Wilks Lambs = 0.92201. The characteristics of homogeneous groups defining the effect of relative humidity outside and relative humidity inside comparison with the CO_2_ and NH_3_ levels were presented in Table 5.

**Table 5:** Characteristics of homogeneous groups defining the effect of relative humidity outside and relative humidity inside comparison with the CO_2_ and NH_3_ levels

Emission level	CO_2_		NH_3_	
	Mean of relative humidity outside	Mean of relative humidity inside	Mean of relative humidity outside	Mean of relative humidity inside
	%			
Low	60.9^a^	64.1^a^	58.5^a^	60.6^a^
Average	86.5^b^	78.9^b^	66.4^b^	68.8^b^
High	-	-	73.1^c^	73.4^c^

^a,b,c^Homogeneous groups

Source: own study

According to Table 6, the increase of relative humidity outside and relative humidity inside caused the increase of CO_2_ and NH_3_ emission. Statistical analysis confirmed the need for reducing the relative humidity inside to limit the CO_2_ and NH_3_ emissions. Considering the whole scope of the conducted studies, it can be noticed that the best conditions for limiting CO_2_ are to reduce temperature and humidity outside and inside of livestock housing. Ammonia emission could be reduced by simultaneously decreasing air humidity and decreasing air temperature. In the case of NH_3_ emission, reduction is necessary to increase the temperature and reduce the humidity inside the building.

**Table 6:** Air exchange rates in cattle barns according to Polish standards

Summer	Winter
350–400 m^3^·h^−1^ for dairy cows	90 m^3^·h^−1^
For cows with higher milk yield, it should be increased by 25%	Deep litter—increase by 50%

Source: own elaboration based on Collective work (2005)

## Discussion

Results derived from our emission experiments were common to other authors (Walczak and Krawczyk) despite weather conditions. In particular, in non-litter cowsheds, higher NH_3_ emissions were observed. A similar situation was described by Zhang who tested ammonia emissions from 11 types of cattle barns, with different floor and manure removing systems and the highest emission was in non-littered cattle barns (Zhang et al., 2005).

Similar results were obtained by a Polish researcher, which calculated ammonia emissions by using model (not measured) from dairy cattle for particular technologies ranging from 6.4 per year for deep litter up to 28.69 kg per year for a slurry system, but these results based only on simply assuming fixed rate of nitrogen losses from manure in livestock buildings (Pietrzak, 2006).

Mosquera et al. (2005) stated that from barns with deep litter, an average ammonia emission was at the level of 13.9 kg per cow and year. It is known from other research tests that ammonia emission from cattle barn with the solid floor was about 50% lower than emission from buildings with the slatted floor (Swierstra et al., 1995). In contrast, research conducted by Baldini shows higher emission factors in cubicles covered with straw (Baldini et al., 2016).

Also, differences of NH_3_ emissions observed between tied and loose housing were observed by Poteko et al. (2019). A mechanical ventilation system was used and ammonia concentration was measured 10 times per hour from exhaust air. In experiments, single data was as average value from measurements during summer season. In our conducted tests for this article, the single result was based on the average from every 5 min during a couple of chosen, representative days in the summer period.

Jungbluth et al. (2001) were conducting NH_3_, CO_2_ and CH_4_ in respiratory chambers and in cattle barn for 50 cows with gravitational ventilation. In building, 27.8 to 50 g·h^−1^ per LU of ammonia emission was obtained. According to results obtained by Koerkamp et al. (1998), ammonia emission from boxed barns was at wide level 987–2001 mg·h^−1^per animal.

There were ammonia emissions tested from beef and dairy cattle barns, and the following results are obtained by Demmers: from a system with slurry, 3.7 kg during 190 days of being inside livestock buildings for beef cattle, and 6 kg during 190 days of being inside livestock buildings for dairy cattle (both indicators based per 500 kg of live weight) (Demmers et al., 1998).

In our research, we obtained higher emissions from all object tested (with bedding and without bedding) compared to other authors’ results.

Table 6 shows the recommended values of the air exchange rate in buildings for cattle in Poland. Only one of the cattle barns tested had ventilation rate below the recommended values.

According to Demmers et al. (1998), the annual NH_3_ emission from litter-free barns was about two times higher than emission from barns with litter. A similar trend was obtained in our research, where the emission from the litter-free system in one of the barns was about 24 kg·year^−1^·LU^−1∙^and for the litter system 12.87 kg·year^−1^·LU^−1^.

## Conclusions

Recently, livestock production significantly increased in Central Europe that involved the need of correction of emission factors. Generally, in Poland, it is utilized emission coefficient elaborated in Northern European countries (UK, DK and NL). In this study, first step was made to present Polish emission factor dedicated especially to summer season conditions.

Although the change of temperature and humidity was not huge, measured values allowed estimating the levels of gas emission in order to carry out the statistical analysis. According to the study, the increase of relative humidity outside and relative humidity inside caused an increase of CO_2_ and NH_3_ emission. A completely different validity occurred in accordance to the measured temperature values, where the increase in temperature could cause the reduction of NH_3_ emission. However, this validity was not confirmed by statistical analysis where the lack of temperature influence on the NH_3_ emission level confirms the value significance level *p* = 0.01184. All other cases of statistical analysis have reached the significance value *p* below the critical level of 0.05. The main conclusion from the research is that ammonia emissions from cattle barns with slurry were higher than from cattle barn bedded with straw, but simultaneously in the night period, both emission levels were comparable.

## Data Availability

Due to confidentiality agreements, supporting data can only be made available to bona fide researchers subject to a non-disclosure agreement. Details of the data and how to request access are available from Kamil Roman at Warsaw University of Life Sciences WULS.
